# Editorial: Molecular Mechanism and Therapeutic Approach to Renal Interstitial Fibrosis

**DOI:** 10.3389/fmed.2022.879927

**Published:** 2022-05-04

**Authors:** Mao-Ting Li, Xiao-Han Tang, Hui Cai, Ai-Hua Zhang, Zhi-Yong Guo

**Affiliations:** ^1^Department of Nephrology, Shanghai Changhai Hospital, Naval Medical University, Shanghai, China; ^2^Department of Pharmacology, Weill Cornell Medical College of Cornell University, New York, NY, United States; ^3^School of Medicine, Emory University, Atlanta, GA, United States; ^4^Nanjing Children's Hospital, Nanjing, China

**Keywords:** epithelial-mesenchymal transition, lncRNA SPANXA2OT1, galectin-3, hydrogen-rich water, native T1 mapping, signal pathway

Renal interstitial fibrosis (RIF) refers to the abnormal deposition of collagen and related proteins in the interstitium of renal cortex, it is a common histological abnormality in all types of renal diseases (1). We have made great strides in the molecular mechanisms and treatment of RIF through a series of explorations.

Epithelial-mesenchymal transition (EMT) is an important initiation link of RIF and previous studies have suggested that EMT has an important role in renal tissue damage repair (2). In nephrolithiasis or crystallize-induced renal injury, EMT occurs in renal tubular epithelial cells at the initial stage of nephrolithiasis formation or the stage of crystallize-induced renal injury, then triggering the process of renal fibrosis (3). Long non-coding RNA (lncRNAs), which are a type of non-coding RNAs, have been shown its importance in the process of organ fibrosis (4). Hu et al. noted that lncRNA SPANXA2OT1 could adsorb miR-204 through sponge-like action, thereby attenuating the inhibitory effect of miR-204 on Smad5 and upregulating Smad5 expression, and is involved in the occurrence and development of EMT in renal tubular epithelial cells. In diabetic kidney disease (DKD), Wang et al. noted that JMJD1A/NR4A1 signaling can regulate the advanced glycation end products (AGEs)-induce EMT in renal tubular epithelial cells. AGEs cause oxidative stress, trigger excessive reactive oxygen species, and promote the production and release of inflammatory cytokines (5). Their continuous stimulation usually causes damage to HK-2 cells in renal tubular epithelial cells, and lead to extracellular matrix (ECM) deposition and abnormal synthesis and degradation of epithelial proteins, further causing epithelial-mesenchymal transition (EMT) and even interstitial fibrosis (6). Overexpression of JMJD1A accelerates the progression of AGEs-induced renal fibrosis by reducing the expression of NR4A1 in HK-2 cells. NR4A1 is closely related to renal fibrosis, and contributes to the occurrence and development of DKD. Therefore, NR4A1 inhibitors could promote the expression of fibrosis-related factors such as VIM, a-SMA in HK-2 cells, and aggravate the process of fibrosis.

In addition to molecular mechanisms, subsequent studies are gradually inclined to clinical. Some important protein molecules have been excavated, which will help to predict the occurrence and development of RIF. Ou et al. noted that the occurrence of RIF may be involved in immune response and related pathways. Plasma Galectin-3 (Gal-3), a multifunctional glycan-binding protein, is widely expressed in immune cells, especially in monocytes/macrophages, which profoundly affect critical macrophage functions, such as phagocytosis and phenotype transition (7, 8). In fibrotic kidney biopsy samples, the level of Gal-3 is upregulated and the differentially expressed genes are mainly enhanced in immune cell activation and the regulation of cell-cell adhesion. Moreover, Gal-3 knockout mice exhibit a decrease in collagen accumulation and resistance to the development of fibrosis in an *in vivo* model (9). Therefore, Gal-3 was suggested to be used as a predictive marker of RIF to identify individuals at risk for renal fibrosis at the earliest possible stage, so as to slow down or even reverse the renal function.

In addition, renal fibrosis is the pathological repair reaction of kidney to chronic injury and is the important process of chronic kidney disease (CKD) progressing to end-stage renal disease (ESRD). Over recent years, obstructive nephropathy caused by urinary calculi has become one of the common factors of CKD. The research related to the treatment and prognosis of RIF is worthy of further exploration. Calcium oxalate (CaOx) is the major component of most kidney stones, which has become the focus of RIF naturally, which can interact with renal tubular epithelial cells and induce oxidative stress and inflammation (10). Excessive oxidative stress and inflammation not only enhance the deposition and retention of CaOx crystals in tubular cells, but also result in the development of fibrosis (11). Therefore, revealing the mechanism of crystalline kidney injury can provide potential targets and pathways for the intervention of renal calculi, and more importantly, provide guidance for the early prevention and treatment of renal calculi and reduce the risk of renal failure. Gao et al. suggested that CaOx crystal could induce inflammatory reaction and oxidative stress through Akt, ERK1/2, and P38 MAPK pathways and affect amino acid metabolism and fatty acids β oxidation, resulting in kidney injury. Besides, according to a series of studies on the treatment of crystalline kidney injury, hydrogen-rich water (HRW), which has been used in the medical field, attracted our attention due to its activities of anti-oxidation, anti-inflammation, anti-fibrosis, and anti-apoptosis (12). The primary advantages of HRW are that it is a portable, easily administered, and safe means of delivering hydrogen. Si et al. noted that HRW administration could alleviate oxidative stress induced by oxalate diet via decreasing the content of MDA, ROS and increase the levels of SOD, GSH-Px and CAT via inhibiting PI3K/AKT, NF-κB pathways. In dependently from this, HRW can ameliorate oxalate-induce renal fibrosis via inhibiting the TGF-β/Smad signaling pathway.

RIF is considered to be a key predictor of renal functional recovery and prognosis in most renal diseases (1). At present, renal biopsy is the gold standard for evaluating RIF. However, due to the extreme shortcomings, its invasiveness, low reproducibility, and the limited size of the collected sample, this approach has not been widely implemented in the clinic (13). Therefore, there is an urgent clinical need for a method for non-invasively evaluating the degree of renal fibrosis. chronic glomerulonephritis (CGN) remains the leading cause of CKD and ESRD in China currently (14). Wu et al. noted that native T1 mapping, a quantitative magnetic resonance imaging (MRI) technique that has been reported to reflect the degree of tissue fibrosis in CGN patients, might serve as an alternative approach to kidney biopsy for detecting renal fibrosis. These is a good correlation between the T1 value and traditional clinical indirect indicators for the evaluation of renal fibrosis, such as SCr, NGAL, CysC, Hb, renal length, eGFR and Hct.

## Author Contributions

M-TL: writing—original draft preparation. Z-YG: writing—review and editing. X-HT, HC, and A-HZ: supervision. All authors contributed to the article and approved the submitted version.

## Conflict of Interest

The authors declare that the research was conducted in the absence of any commercial or financial relationships that could be construed as a potential conflict of interest.

## Publisher's Note

All claims expressed in this article are solely those of the authors and do not necessarily represent those of their affiliated organizations, or those of the publisher, the editors and the reviewers. Any product that may be evaluated in this article, or claim that may be made by its manufacturer, is not guaranteed or endorsed by the publisher.
